# Influence factors associated with health risk behaviors of middle school students in the poverty area of China: An observational study

**DOI:** 10.1097/MD.0000000000029922

**Published:** 2022-08-19

**Authors:** Gaoqi Ge, Chaoji Huangfu, Min Ge, Yuxia Gao, Nan Tang

**Affiliations:** a Henan Provincial People’s Hospital, Zhengzhou, Henan Province, China; b Nursing School of Jilin University, Changchun, Jilin Province, China; c Center for Disease Control and Prevention, Lanzhou, Gansu Province, China; d Puyang City People’s Hospital, Puyang, Henan Province, 457000, China; e Center for evidence-based nursing, School of Nursing, Lanzhou University, Lanzhou, Gansu Province, China.

**Keywords:** demographic factors, family care, health risk behaviors, middle school students, social support

## Abstract

We aim to investigate the status and influence factors of health risk behaviors among middle school students and explore the relationship between social support, family care, and the health risk behaviors.

The study was conducted in 3 middle schools in the Fan county located in the Puyang city. Independent measures were applied to assess adolescent health risk behaviors, perceive social support, and family care. Multiple regression analysis was used to analyze the main factors that affect adolescent health risk behaviors.

The total scores of health risk behaviors were 53.87 ± 9.97, and all kinds of health risk behaviors were very common. The highest score was health-compromising (2.45 ± 0.43), and the lowest score was unprotected sex behaviors (1.07 ± 0.28). Multiple regression analysis showed that sex (*P* < .001), grade (*P* < .001), parent relationships (*P* < .001), father’s occupation (*P* = .035), mother’s education level (*P* = .011), social support (*P* < .001), affection (*P* < .001), and growth (*P* = .003) were the main factors of health risk behaviors, accounting for 25.3%.

The health risk behaviors among middle school students in Fan county should attract the attention of education administration, schools, and parent due to the varied influencing factors. Related interventions should be conducted to reduce the severity and frequency of adolescent health risk behaviors and protect the health and growth of adolescents. In order to better analyze the health risk behaviors of middle school students, we will incorporate more influencing factors and carry out further causal analysis in the future.

## 1. Introduction

Middle school students are in early adolescence and are the first step in the transition from school age to adulthood. They are curious and full of doubts about the world, and they have the courage to try new things. With the continuous development and maturity of independence and self-awareness of middle students, they want to escape parents’ supervision and are eager to better integrate into the school and establish their own friends’ circle.^[1]^ However, they have not established mature values, so they cannot correctly estimate the potential consequences caused by health risk behaviors.

Adolescent health risk behaviors severely affect the health and body conditions of teenagers and may even affect their health in adulthood period.^[2]^ These behaviors may cause severe social problems.^[3]^ American Adolescent Health Risk Behavior Monitoring Questionnaire has been used commonly to investigate the health status and health risk behavior of teenagers. Three large-scale surveys aimed at studying the adolescent health risk behaviors of Chinese middle school students have been conducted in 1996, 2005, and 2008, respectively.^[4]^ However, these studies mainly focus on the students in the developed urban areas of China. Study of adolescent health risk behaviors in terms of middle students in the poor areas of China is very rare.

The teenagers in the poor areas of China are a special population. For the poor areas of China, the average income and educational level of people are much lower than that those of urban areas. These teenagers face a myriad of barriers to health, including limited resources and access to health care, low socioeconomic status, and low educational attainment. Individuals who face these inequalities are more likely to have low health literacy.^[5]^ Most adults in the poor area choose to work in the developed cities for a higher income. Due to the limitation of housing and education in the developed cities, their children are left in hometown and live with grandparents. Compared with students in the urban areas, the students in the poverty areas lack effective parental supervision. Therefore, these teenagers may be susceptible to health risk behaviors.

In the present study, middle school students in the Fan county, a poverty-stricken county, were selected. We investigated the status of health risk behaviors among middle school students in the Fan county. Then, the types of demographic data that influence the adolescent health risk behaviors were analyzed. Meanwhile, we studied the correlation of health risk behaviors with social support and family care. Finally, the main influencing factors of middle school students’ health risk behaviors were analyzed.

A previous study indicated that adolescent health risk behaviors in China are more serious than our imagination.^[6,7]^ Unfolding the adolescent health risk behaviors earlier is necessary to analyze relevant influencing factors and is beneficial to take suitable interventions to reduce the incidence of health risk behaviors.

## 2. Materials and Methods

### 2.1. Participants

All methods were performed in accordance with the relevant guidelines and regulations, and this study was approved by the Ethics Committee of the school of Nursing, Jilin University (2017-04-28). The survey was conducted from June to July 2017. The survey was authorized by the main administrators of school. Letters of informed consent were sent to students or their parents/guardians. All participants and their parents agreed to join this survey. The use of questionnaires was authorized by related authors.

**Figure 1.**
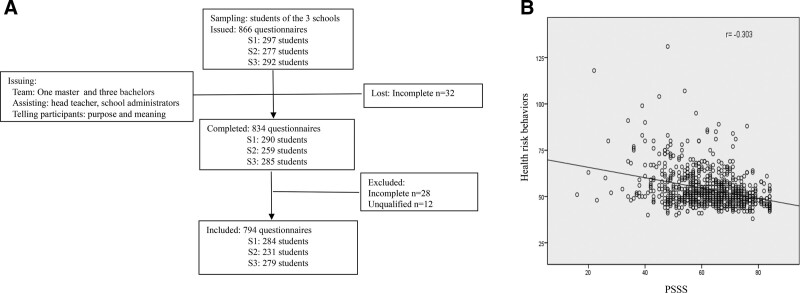
(A) Data collection procedure. (B) The relationship between PSSS and health risk behaviors was analyzed. PSSS = Perceived Social Support Scale.

The study applied 2-stage cluster sampling (Fig. 1A). In the first stage, 3 schools in the Fan county were selected randomly with lottery method. In the second stage, 3 classes from every grade of these schools were selected randomly with lottery method. All students in the randomly selected classes at the schools took part in the survey.

A total of 866 questionnaires were distributed, and 794 were returned and completed (response rate: 91.7%). The samples consist of 353 boys (45%) and 441 girls (55%), ranging in age from 12 to 17 years (mean age being: 14.38, standard deviation = 1.009). Overall, the students from grade 7, grade 8, and grade 9 account for 30.1%, 40.7%, and 29.2%. The study population contained 377 (48%) left-behind teenagers, who live with their grandparents. 7.1% of the students do not have siblings.

Around 56.9% of students reported low/moderate success, and >75% of students claimed having good relationship with their parents. More than half parents of students just completed secondary school. The monthly income of them mainly ranged from 436 to 726 dollar (The exchange rate on the day of the survey (May 24, 2017) was: 1 dollar = 6.887 yuan).

### 2.2 Instruments

#### 2.2.1. Demographic information.

We referred to the relevant references^[8,9]^ and formed a questionnaire of demographic information, which included general questions such as age, grade, sex, race, parental education, parental occupation, parental relationship, monthly income, school success in the last 6 months, etc.

#### 2.2.2. Adolescent Health-Related Risk Behavior Inventory.

The Adolescent Health-related Risk Behavior Inventory designed by Wang et al^[10]^ is a method to assess health risk behavior, consisting of 38 items with responses in the form of a 5-point Likert scale ranging from 1 (never) to 5 (frequently). The tool includes 6 dimensions, which are focused on the level and frequency of suicide, self-injury, health-compromising, aggression and violence, rule breaking, smoking and drinking, and unprotected sex behavior. In these measures, higher scores indicate higher level and frequency of health risk behaviors. The scale has been found to have a good internal consistency reliability with a Cronbach α of 0.90. The Adolescent Health-related Risk Behavior Inventory has been reported to have good test-retest reliability with the Cronbach α at 0.810 in this study.

#### 2.2.3. Perceived Social Support Scale.^[11]^

The scale is a measure of perceptions of social support, consisting of 12 items with responses in the form of a 7-point Likert scale ranging from 1 (strongly disagree) to 7 (strongly agree). The questionnaire is focused on family support, social support and other support. Higher scores indicate higher social support. In this study, we revised the “leaders, relatives, and colleagues” in other support items into “teachers, classmates, and relatives.” The Cronbach α coefficient of the reliability of this scale was calculated as 0.88, and a Cronbach α of 0.872 for this scale was calculated in this study, which is considered indicative of relatively high internal consistency.

#### 2.2.4. Family APGAR Index.^[12]^

The scale is a measure of perceptions of family care, consisting of 5 items that correspond to 5 dimensions (adaptation, partnership, growth, affection, and resolve). Youth rate each item on a 3-point Likert scale (0 = not at all; 2 = very much), and higher scores indicate family function is good. Lv et al^[13]^ applied a weighted Kappa to the scale and every dimension to test the reliability. The results indicated that the Kappa (w) values of the other 4 dimensions are all >0.7 except for fitness, and the total scale’s Kappa (w) is >0.4, indicating that the questionnaire has good consistency. Cronbach α for this scale was 0.780 in this study, suggesting good internal consistency.

#### 2.2.5. Data collection and procedures.

Questionnaires were distributed and answered face-to-face. The research team contacted main administrators of 3 schools and determined the investigation time. The questionnaires were distributed by the research team in the class. Before answering the questionnaires, the researchers explained the purpose and means of the survey and the procedures. The students were asked to focus on their own response without discussion with other students. If there was a problem, the investigator should explain. Questionnaire should be completed and returned within 40 minutes. After receiving the questionnaire, the investigators were responsible for checking the integrity and effectiveness of the questionnaire and eliminating the invalid questionnaire. A pilot study was conducted to test the reliability of questionnaire before being used in this study. The pilot study showed that the total instruments had good internal consistency, and students could easily understand the questions. To protect student privacy, the anonymous and self-completed questionnaires were distributed by the researchers in the absence of the teachers.

### 2.3. Statistical analysis

Data were entered using EpiData 3.1, and all statistical analyses were performed using the Statistical Package for Social Sciences (SPSS) version 17.0. We conducted descriptive analyses for sociodemographic characteristics and health risks. *T* tests were used to compare the composite scores on health risk behaviors in different groups. Spearman correlations were also conducted between health risk behaviors and social support and family care. A standard multiple regression analysis was used to predict main factors of health risk behaviors. Differences were considered statistically significant at a *P* value of <.05.

## 3. Results

### 3.1. Status of health risk behaviors among middle school students in Fan county

The standardized score of each dimension was obtained by dividing the dimension score by the number of items in the dimension. The data showed that the scores of all kinds of health risk behaviors among students in Fan County from high to low were health-compromising, aggression and violence, rule breaking, suicide and self-injury, smoking and drinking, and unprotected sex behaviors (Table 1).

**Table 1:** Scores of the health risk behaviors (N = 794).

	Unstandardized score (x ± s)	Standardized score (x ± s)
Health-compromising behaviors	12.23 ± 2.13	2.45 ± 0.43
Aggression and violence	13.84 ± 3.80	1.38 ± 0.38
Rule breaking	9.59 ± 2.43	1.37 ± 0.35
Suicide and self-injury	5.89 ± 1.98	1.18 ± 0.40
Smoking and drinking	6.95 ± 2.14	1.16 ± 0.36
Unprotected sex	5.37 ± 1.38	1.07 ± 0.28
Total score of health-related risky behaviors	53.87 ± 9.97	1.42 ± 0.26

Unstandardized scores: total score of the original scale and scores of each dimension. Normalized score: total score divided by item number score, each dimension score divided by the number of corresponding dimension items.

x ± s = mean ± standard deviation.

### 3.2. Sociodemographic factors and health risk behaviors

There were significant differences in the health risk behaviors among the students in the 14 variables such as grade, sex, parental education, parental occupation, and school success in the last 6 months (Table 2). The health risk behavior scores of students in the 7th grade were significantly higher than students from other grades. Students whose parents are government staff always had lower scores than other occupations, and students whose parental education comprised a university or college degree or above always had lower scores than other education levels. Males’ health risk behavior scores were significantly higher than females’ scores (*t* = 6.598, *P* = .000). There were significant differences in the health risk behaviors among students comparing their family monthly income (*t* = 3.533, *P* = .007), and the score of health risk behaviors in ≥10,000 level was higher than other levels. The health risk behavior scores were higher among the students who were left behind by their parents (*t* = 2.333, *P* = .020). Students who belonged to large/nuclear families had significantly lower scores than those in single/remarried families (*t* = –2.566, *P* = .015). Students who had been living with both parents for a long time were more likely to have lower scores than those who had not living with both parents for a long time (*t* = –3.374, *P* = .001). Students whose parental relationship was good were at a significantly lower risk than those whose parental relationship was general/poor (*t* = –3.978, *P* = .000), namely, with regard to the relationship with the father (*t* = –4.356, *P* = .000) and the relationship with the mother (*t* = –3.978, *P* = .000). Students who reported high school success tended to have lower scores of health risk behaviors (*t* = –3.999, *P* = .000). Age, character, and sibling status were not significant influencing factors in health risk behaviors among these students.

**Table 2:** Comparisons of student demographics and the characteristics related to health risk behaviors (N = 794).

	N (%)	Health risk behaviors score (x ± s)	*t*/F	*P* value
Grade			18.581	.000**
7th	239 (30.1)	56.77 ± 10.87		
8th	323 (40.7)	53.54 ± 10.19		
8th	232 (29.2)	51.33 ± 7.71		
Gender			6.598	.000**
Boys	353 (44.5)	56.49 ± 11.15		
Girls	441 (55.5)	51.77 ± 8.36		
Age (yr)			0.647	.524
12–13	145 (18.3)	54.64 ± 10.76		
14–15	543 (68.4)	53.61 ± 9.40		
16–17	106 (13.4)	54.12 ± 11.63		
Having sibling			0.184	.854
No	742 (93.5)	53.85 ± 10.02		
Yes	52 (6.5)	54.12 ± 9.35		
Parental occupation				
Father			6.479	.000**
Government staff	69 (8.7)	48.97 ± 5.98		
Worker	156 (19.6)	54.90 ± 8.93		
Self-employed	122 (15.4)	52.16 ± 9.97		
Farmer	158 (19.9)	54.77 ± 11.24		
Unemployment/other	289 (36.4)			
Mother			5.972	.000**
Government staff	77 (9.7)	50.35 ± 7.57		
Worker	89 (11.2)	53.64 ± 7.76		
Self-employed	118 (14.9)	51.55 ± 8.29		
Farmer	236 (29.7)	54.50 ± 9.49		
Unemployment/other	274 (34.5)	55.39 ± 11.75		
Parental education				
Father			5.760	.001**
Completed primary school or below	114 (14.4)	54.94 ± 10.82		
Completed secondary school	502 (63.2)	54.27 ± 10.06		
Vocational education/high school	96 (12.1)	54.11 ± 9.08		
University or college degree or above	82 (10.3)	49.63 ± 8.16		
Mother			4.648	.003**
Completed primary school or below	216 (27.2)	54.40 ± 9.44		
Completed secondary school	438 (55.2)	54.18 ± 10.45		
Vocational education/high school	78 (9.8)	54.26 ± 10.38		
University and college degree	62 (7.8)	49.37 ± 6.22		
Monthly income (USD)			3.533	.007**
≤145	79 (9.9)	54.23 ± 9.71		
146–436	306 (38.5)	54.43 ± 10.19		
437–726	255 (32.1)	53.82 ± 9.72		
727–1452	129 (16.2)	51.50 ± 8.46		
>1452	25 (3.1)	58.60 ± 14.88		
Left-behind children			2.333	.020*
Yes	377 (47.5)	54.73 ± 10.48		
No	417 (52.5)	53.09 ± 9.43		
Family structure			–2.566	.015*
Big family/nuclear family	764 (96.2)	53.66 ± 9.84		
Single family/remarried family	30 (3.8)	59.27 ± 11.81		
Living with both parents for a long time			–3.374	.001**
Yes	475 (59.8)	52.90 ± 9.86		
No	319 (40.2)	55.32 ± 9.98		
Relationship with father			–4.356	.000**
Good	613 (77.2)	52.94 ± 9.30		
General/poor	181 (22.8)	57.00 ± 11.46		
Relationship with mother			–3.978	.000**
Good	697 (87.8)	53.23 ± 9.39		
General/poor	97 (12.2)	58.49 ± 12.57		
Parental relationship			–5.704	.000**
Good	643 (81.0)	52.73 ± 9.04		
General/poor	151 (19.0)	58.72 ± 12.13		
School success			–3.999	.000**
High	342 (43.1)	52.30 ± 8.86		
Low/moderate	452 (56.9)	55.06 ± 10.59		
Own character			1.624	.198
Introverted	175 (22.0)	52.93 ± 8.41		
Neutral	365 (46.0)	53.76 ± 9.41		
Extroverted	254 (32.0)	54.67 ± 11.59		

The exchange rate on the day of the survey (May 24, 2017) was: 1 dollar = 6.887 yuan.

x ± s = mean ± standard deviation.

* *P* < .05.

** *P* < .01.

### 3.3. Associations of health risk behaviors with social support and family care

The results of this study showed that there was a negative correlation between health risk behaviors and social support (*r* = –0.303), such as friend support (*r* = –0.251), family support (*r* = –0.280), and other support (*r* = –0.270). There was also a negative correlation between health risk behaviors and family care (*r* = –0.386), such as adaptation (*r* = –0.290), partnership (*r* = –0.273), growth (*r* = –0.263), affection (*r* = –0.341), and resolve (*r* = –0.272; Table 3). As described previously,^[14]^ 0.1< “*r*” < 0.3 indicates a small/weak correlation, 0.3 < “*r*” < 0.5 indicates a medium/moderate correlation, “*r*” >5 indicates a large/strong correlation. The results of our study showed that there was a moderate correlation between health risk behaviors and social support, and weak correlation between health risk behaviors and friend support, family support, and other support. There was also a moderate correlation between health risk behaviors and family care, affection. The correlations between health risk behaviors and adaptation, partnership, and growth were weak.

**Table 3:** The correlation between health risk behaviors and social support and family care.

	Friend support	Family support	Other support	PSSS	Adaptation	Partnership	Growth	Affection	Resolve	APGAR
*r*	–0.251	–0.280	–0.270	–0.303	–0.290	–0.273	–0.263	–0.341	–0.272	–0.386
*p*	*P* < .01	*P* < .01	*P* < .01	*P* < .01	*P* < .01	*P* < .01	*P* < .01	*P* < .01	*P* < .01	*P* < .01

“*r*” is the correlation coefficient. When –1 ≤ *R* < 0, there is a negative correlation between variables. When 0 < *R* ≤ 1, there is a positive correlation between variables. The greater the value of “*r*,” the better the correlation.

PSSS = Perceived Social Support Scale.

### 3.4. Predictors of health risk behaviors

We used multiple regression to analyze the predictors of health risk behaviors. The results indicated that grade, sex, parental relationship, father’s occupation, mother’s education level, social support, affection, and growth were significant predictors of health risk behaviors. The full model was significant (F = 14.434; *P* = .000) and accounted for 25.3% of the variance in health risk behaviors among students (Table 4). The result showed that sex was the most important factor associated with health risk behaviors, and the score was higher in boys (*β =* –0.200, *t* = –6.342, *P <* .001). Higher perception of social support, affection, and growth indicates lower score of health risk behaviors (*β =* –0.161, *t* = –4.514, *P <* .001; *β =* –0.161, *t* = –4.419, *P <* .001; *β =* –0.104, *t* = –2.930, *P <* .01). In addition, the relationship between Perceived Social Support Scale and health risk behaviors was analyzed. Strong negative correlation between Perceived Social Support Scale and health risk behaviors was observed (Fig. 1B).

**Table 4:** Results of the multiple regression analysis of the predictors of health risk behaviors.

Model	Unstandardized coefficients		Standardized coefficients	*t*	Sig.	Tolerance	VIF
	B	Std. error	Beta				
(Constant)	77.202	2.784		27.730	.000		
Gender	–4.013	0.633	–0.200	–6.342	.000	0.946	1.057
Parental relationship	3.312	0.834	0.130	3.974	.000	0.874	1.144
Grade	–1.732	0.421	–0.134	–4.112	.000	0.889	1.125
Father occupation (farmer)							
Government staff	–4.164	1.970	–0.118	–2.114	.035	0.304	3.291
Mother education (completed primary school or below)							
Vocational education /high school	3.304	1.294	0.099	2.553	.011	0.631	1.586
PSSS	–0.135	0.030	–0.161	–4.514	.000	0.743	1.345
Affection	–-2.402	0.544	–0.161	–4.419	.000	0.706	1.417
Growth	–-1.637	0.559	–0.104	–2.930	.003	0.749	1.335

*R* = 0.521, *R*^*2*^ = 0.272, *R*^*2*^(adj) = 0.253, *F* = 14.434, *P* = .000.

## 4. Discussion

Adolescents easily develop multiple types of health risk behaviors, meaning that health risk behaviors are not a single occurrence but are related to each other. Previous studies mainly focused on the single behavior status and influence factors. The main analysis of various dangerous behaviors is qualitative (yes/no) and lacks the severity comparison of different health risk behaviors. Through Adolescent Health-related Risk Behavior Inventory, we can identify high-risk behaviors in various types of health risk behaviors and then make some targeted interventions to reduce the severity and frequency of adolescent health risk behaviors, promoting students to grow in a healthy way.

This study indicates that health risk behaviors among middle school students in Fan county are severe. The health-compromising behavior in this study includes unhealthy eating behavior (no breakfast, overeating, and excessive dieting) and lack of participation in sports activities. Unhealthy eating habits affect the student’s appearance in school and increase the risk of some diseases such as anemia,^[15]^ obesity,^[16]^ cardiovascular disease,^[17]^ irritable bowel syndrome,^[18]^ and breast cancer.^[19]^ Local education department should pay more attention to this issue, carrying out relevant health education and providing a comprehensive and balanced diet for young people in schools.

Previous studies have found that gender was significantly related to adolescent health risk behaviors.^[20–22]^ The prevalence of aggression and violence,^[20]^ rule breaking,^[21]^ smoking, and drinking^[23]^ was significantly higher in boys than girls. Meanwhile, the prevalence of health-compromising, suicide, and self-injury was different in boys and girls.

We demonstrated that students of higher grades had lower severity of the health risk behavior, which is consistent with previous studies regarding suicide,^[24]^ bullying,^[25]^ and violent behaviors.^[26]^ The students with lower grades are younger and easily tempted. However, they cannot clearly foresee the consequence of risk behaviors. Students whose parental relationship was good had a lower risk of health risk behaviors. The period of junior high school is an important period for shaping personality and students could be directly affected by the growth environment. Good parental relationships create a good family atmosphere, which helps teenagers to deal with problems, communicate with others, and promote their better integration into society. Conversely, students who have bad parental relationship or are frequently exposed to dangerous behaviors are more likely to have health risk behaviors.

Adolescents whose father’s occupation is government staff reported lower scores of health risk behavior. Government staff involve civil servants, teachers, medical staff, academics, etc. These people generally have high levels of education and pay more attention to the mental and physical health of teenagers. Several studies have proved that high levels of mother’s education appeared to be a significant protective factor against unsafe sex behaviors,^[27]^ rule-breaking behaviors,^[28]^ bullying behaviors, and smoking behaviors.^[29]^ This study showed that students whose mother’s education included vocational/high school had a significantly higher risk for developing health risk behaviors than students whose mother’s education included completed primary school or below. Mothers with lower level education are not easy to find work, so they can only stay at home to look after children and the elderly. This may increase the supervision to adolescents, which effectively reduce the incidence of adolescent health risk behaviors.^[30,31]^ Mothers with a higher level of education have the ability to work and the need to bear the family burden. Therefore, the scores of health risk behaviors of their children are higher.

The social support score increased with a decreasing health risk behavior score. Higher level of social support leads to higher level of mental health, stronger ability to deal with problems in life, and better resistance to various kinds of bad temptations around them. It is suggested that a certain level of support should be given to students by the parents, teachers, and peers to reduce the occurrence of health risk behaviors in junior high school students. In this study, higher perception of affection and growth indicates lower score of health risk behaviors. Care and love contribute to intimate and harmonious relationship between family members, so the potential problems of children can be quickly found by parents.

In this study, age was not associated with health risk behaviors, which is not consistent with the findings of several other reports.^[32,33]^ In a poverty area such as Fan county, adolescents have to bear the family burden earlier for a better life, so the influence of age on the lifestyle and behaviors is small. Meanwhile, there are relatively few temptations in such kind of poverty areas, and the living environment of students of different ages are almost the same, so there are no significant differences in the scores of health risk behaviors. Further research is needed to understand the phenomenon in light of socioeconomic and cultural backgrounds.

This study investigates the severity and frequency of concurrent health risk behaviors and their association with demographic and socioeconomic factors among adolescents in the poverty area of middle China, but there are several limitations. First, the study is a cross-sectional study, from which we cannot infer causal relationships between social support, family care, demographic factors, and students’ health risk behaviors. Second, influenced by the sampling method and the socioeconomic culture of the region, findings from this study might not be generalizable to middle school students in other poverty areas in China. Third, this study is based on self-reports, which may be subject to recall bias and social desirability, and there may be cases of underreporting. The questionnaire was distributed 5 years ago, which is a limitation of this research. With the advancement of China’s poverty alleviation work, F County was removed from the name of national-level poverty-stricken county in February 2020. However, with the outbreak of the coronavirus disease 2019, most migrant workers were unable to go out to work and their family economic income was affected to a certain extent.^[34,35]^ In the future, we would investigate the health risk behaviors of adolescents in the postepidemic era to clarify the impact of economic level and left-behind status on adolescent health risk behaviors.

## 5. Conclusions

Male, lower grade, and having a poor parental relationship indicate a higher score of health risk behaviors. Therefore, teachers and government administrators should focus on these students, identifying bad behaviors in time, and help them make corrections. The results of this study showed that there was a significant negative association between health risk behaviors and social support and family care. The score of health risk behaviors decreased markedly with increasing perception of affection and growth and social support. It is suggested that we should encourage families, teachers, and peers to give certain support and care to junior high school students, reducing the occurrence of health risk behaviors, and promoting the healthy growth of them. In order to better analyze the health risk behaviors of middle school students, more influencing factors should be included in the future, and long-term longitudinal tracking should be carried out to analyze the causal relationship between relevant factors and health risk behaviors.
